# Return to work after carpal tunnel release surgery: a qualitative interview study

**DOI:** 10.1186/s12891-019-2638-5

**Published:** 2019-05-22

**Authors:** Lisa Newington, Charlotte Brooks, David Warwick, Jo Adams, Karen Walker-Bone

**Affiliations:** 1 Arthritis Research UK – MRC Centre for Musculoskeletal Health and Work, MRC Lifecourse Epidemiology Unit, Faculty of Medicine, University of Southampton, Southampton General Hospital (MP 95), Tremona Road, Southampton, SO16 6YD UK; 2 Hand Therapy, Chelsea and Westminster Hospital NHS Foundation Trust, 369 Fulham Road, London, SW10 9NH UK; 3 School of Health Sciences, Faculty of Environmental and Life Sciences, University of Southampton, University Road, Southampton, SO17 1BJ UK; 4 Therapy Department, University Hospital Southampton NHS Foundation Trust, Southampton General Hospital, Tremona Road, Southampton, SO16 6YD UK; 5 Faculty of Medicine, University Hospital Southampton NHS Foundation Trust, Southampton General Hospital, Tremona Road, Southampton, SO16 6YD UK

**Keywords:** Carpal tunnel release, Carpal tunnel syndrome, Work, Return to work, Patient experience, Qualitative interviews

## Abstract

**Background:**

Carpal tunnel syndrome is a common nerve compression disorder which affects hand sensation and function. Carpal tunnel release surgery (CTR) is frequently performed to alleviate these symptoms. For many CTR patients, surgery occurs during their working lifetime, but there is currently no evidence-based guidance to inform clinicians or patients when it might be safe to return to different types of work afterwards. The aim of this qualitative study was to explore the return to work experiences of patients who had recently undergone CTR.

**Methods:**

Semi-structured 1:1 interviews were conducted with a subgroup of participants recruited to a multi-centre prospective cohort study. Interviewees were purposely selected to represent a range of demographic, clinical and occupational characteristics. All had recently undergone CTR and had returned to work. Interviews were audio recorded, transcribed verbatim and analysed using the framework method. Participants were recruited until data saturation was achieved.

**Results:**

Fourteen participants were interviewed: 11 women (median age 49 years, range 27–61) and 3 men (age range 51–68 years). Three key themes were identified. Theme 1 centred on the level of functional disability experienced immediately after surgery. There was an expectation that CTR would be a ‘minor’ procedure, but this did not match the participants’ experiences. Theme 2 explored the desire for validation for the time away from work, with participants recalling a need to justify their work absence to themselves as well as to their employers. Theme 3 focused on the participants’ reflections of handing their return to work and function, with many reporting uncertainties about what constituted appropriate activity loads and durations. There was a desire for specific information relating to individual work roles.

**Conclusion:**

Individual return to work decision-making was largely influenced by the recommendations received. According to the views of participants, clinicians may be able to prepare patients better pre-operatively, especially with respect to function in the immediate post-operative period and by providing return to work guidance that can be tailored for individual work roles.

**Electronic supplementary material:**

The online version of this article (10.1186/s12891-019-2638-5) contains supplementary material, which is available to authorized users.

## Background

Carpal tunnel syndrome (CTS) is a common peripheral nerve entrapment syndrome, occurring when the median nerve becomes compressed within the carpal tunnel. Symptoms include an unpleasant tingling or reduced sensation in the radial digits, and weakness of the thenar muscles. This reduces manual dexterity and often disturbs sleep [1]. As the peak incidence for CTS occurs during the working lifetime [2], both the symptoms from, and treatment of, CTS may affect people in the workplace. The US National Health Interview Survey found that among current/recent workers, the life-time prevalence of CTS was 6.7% [3]. Specific CTS incidence or prevalence data for UK workers were not found.

The first line of treatment for CTS involves wrist splints and/or corticosteroid injection, however in more severe cases, or when non-operative treatment has failed, carpal tunnel release (CTR) surgery is recommended [4]. More than 90,000 CTR procedures are expected annually by 2020 in the English NHS alone [5], but there is currently no evidence-based guidance advising when it might be safe to return to functional activities, including work, after CTR. Our recent systematic review of the duration of work absence after CTR highlighted considerable variation in reported return to work times [6]. Across 56 included studies, mean time to return to work ranged from 4 to 168 days (24 weeks). Unfortunately, occupational characteristics were only reported by a small minority of studies, but those which did suggested longer durations of work absence in the following scenarios: employed, rather than self-employed workers; part-time, rather than full-time workers; manual rather than non-manual workers; and for those receiving workers’ compensation. Prognostic factors associated with earlier return to work have been reviewed by Peters et al. and included an expectation or desire for fewer days off work, lower pain anxiety and a work role that was unaffected by CTS [7].

To date, the research in this field has focused on quantitative measures of return to work with little attention given to patients’ experiences. We were interested in exploring patients’ perspectives of returning to their work after CTR and in identifying the factors that influenced this return to work experience.

## Methods

### Study design and research team

This semi-structured qualitative interview study was nested within an existing NIHR-funded cohort study, known as REACTS (Return to Employment After Carpal Tunnel release Surgery, NIHR DRF-2015-08-056). The lead author (LN) was a practising physiotherapist and PhD candidate and the research team also comprised academic and clinical academic healthcare researchers in the fields of rheumatology, occupational therapy and hand surgery. This research was supported by a group of patient advisors who had all previously undergone CTR.

### Participants and recruitment

Interviewees were purposively recruited from the REACTS study. REACTS participants had been recruited from 16 sites across England and Wales and eligibility criteria were: referred for CTR, aged ≥18 years, routinely working in paid employment for ≥20 h per week and planning to return to work after CTR, no previous CTR to either hand. The sampling frame for the nested interview study took into account age, sex, type of work and work contract, study site and duration of work absence after CTR. Using this purposive sampling frame, REACTS participants were invited to take part in an interview after completing their final REACTS study questionnaire (approximately 3 months after CTR).

### Data collection

Participant experiences of returning to work after CTR were explored using semi-structured one-to-one interviews conducted by the lead author (LN). The interviews were either conducted face-to-face or by telephone, according to participant preference, and were audio recorded and transcribed verbatim. All transcripts were checked against the original audio. The interview guide was developed and piloted with the patient advisory group and contained questions concerning: hand/wrist symptoms; return to work decision-making; and the individual’s experience of returning to work. The full interview guide is provided in Additional file 1.

Recruitment continued until the research team were confident that data saturation was achieved, therefore data collection and the initial phases of analysis occurred concurrently. The definition of data saturation was two-fold to encompass both sampling and analytical saturation [8]. The first phase of saturation occurred when the interviewer began hearing the same comments repeated by different interviewees [9], and this was confirmed by the second phase of saturation when no new codes were identified during the data analysis [10].

### Analysis

Data were managed and analysed using the Framework Method [11]. The first two transcripts were read and re-read and preliminary codes were identified independently by the research team. An additional transcript was independently reviewed and coded by LN and CB. The codes were then discussed and a coding framework was created and applied line by line to all transcripts by the lead author (LN) using NVivo software (Version 11, QSR International Ltd). Where new codes were identified in later transcripts, these were logged and discussed with the research team to ensure agreement and were applied to all transcripts, where appropriate. Analytical ideas were noted and discussed with the research team throughout this initial coding phase and were subsequently explored using the matrices function in NVivo. The coded text was summarised by the lead author to create a series of framework matrices illustrating the key points for each passage of text, which could be viewed across participants and coding topics. These charted framework matrices were reviewed by the research team and organised to illustrate the key themes discussed by interviewees and to identify situations in which there were obvious differences. As the developing themes were explored in detail, sub-themes were created to illustrate these differences. The themes were reviewed by the patient advisory group and their comments incorporated into the final draft.

## Results

### Participants

Fourteen interviews were completed to a point where the research team agreed that data saturation had been achieved. These individuals were recruited from a total of 31 invitation letters. Variation was achieved for all demographic characteristics included in the sampling frame (Table 1). Eight healthcare facilities were represented by the interview participants, including NHS primary and secondary care services and private healthcare settings across southern and central England. All interviews took place between August 2017 and June 2018.

**Table 1:** Participant demographic and occupational characteristics

Pseudonym	Sex	Age category (years)	Healthcare setting	Type of work contract^a^	Work role	Available sick pay	Dominant hand	Side of CTR	Post-operative work absence (days)		Interview timing (days after surgery)
									Expected	Actual	
Jill	F	51–60	NHS 2	Employed	Sales assistant	Unsure	Right	Right	14	21	119
Debbie	F	51–60	NHS 1	Employed	Nurse	> 6 months	Right	Right	21	21	152
Alan	M	51–60	NHS 1	Self-employed	Maintenance	< 1 week	Right	Right	7	7	151
Sarah	F	61–70	NHS 1	Self-employed	Stable owner	> 6 months	Right	Right	NR	0	123
Peter	M	51–60	NHS 2	Employed	Mechanic	< 1 week	Right	Left	21	16	130
Emma	F	51–60	NHS 2	Employed	Optician	1–6 months	Both	Left	42	42	115
George	M	61–70	NHS 2	Self-employed	Gardener	< 1 week	Left	Right	21	14	155
Helen	F	51–60	NHS 1	Employed	Nurse	> 6 months	Right	Right	21	8	149
Fiona	F	21–30	NHS 1	Employed	Animal technician	1–6 months	Right	Right	21	21	160
Donna	F	31–40	NHS 2	Employed	Police officer	1–6 months	Right	Left	14	42	118
Charlotte	F	41–50	NHS 2	Employed	Postal worker	1–6 months	Right	Right	21	98	119
Vicky	F	41–50	Private	Employed	Secretary	1–6 months	Right	Right	7	4	114
Amanda	F	41–50	Private	Employed	Administrator	> 6 months	Right	Right	14	6	155
Alison	F	41–50	NHS 2	Zero hours contract	Carer	< 1 week	Right	Right	21	28	94

^a^All participants listed as employed reported having a permanent work contract

*CTR* carpal tunnel release, *NR* not reported, *F* female, *M* male, *NHS 1* NHS primary care, *NHS 2* NHS secondary care, *private* private healthcare setting

The majority of interviewees were female (11/14) and the median age was 51 years. Seventy-one percent were employed, although self-employed workers (*n* = 3) and those on zero hours contracts (*n* = 1) were also represented in similar proportions to those found across the whole REACTS cohort. Interviewees worked in a range of different industries with varied occupational roles. Participant demographics are illustrated in Table 1.

Thirteen interviews were conducted by telephone and one was conducted face to face. The mean interview duration was 27 min, and the range was 16–48 min. The median duration between CTR and interview was 127 days (range 94–160).

### Key themes

**Fig. 1:**
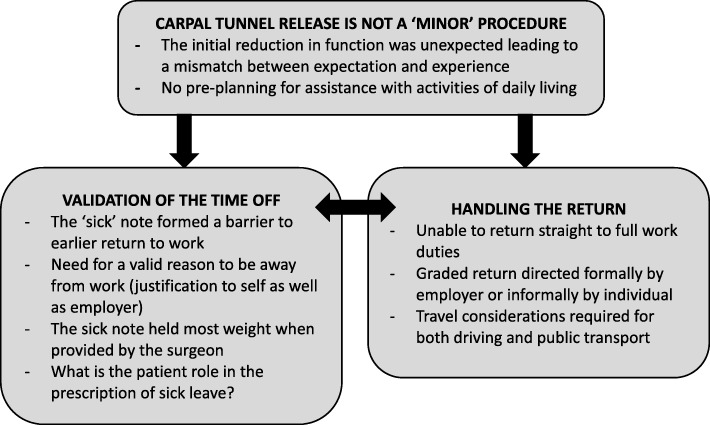
Key themes identified in the return to work experiences of patients after carpal tunnel release

Three key themes were identified from the interview texts, providing insight to the personal experience of returning to work (Fig. 1). The first theme centred on a perceived lack of preparedness for functional difficulties experienced in the immediate post-operative period: *CTR is not a ‘minor’ procedure*. The second theme explored the desire for *validation for time off* work, while the third encompassed the participants’ reflections on *handling the return* to work and function*.* The three themes are explored below using illustrative quotes from the interviewees, presented with pseudonyms. Additional quotes to support each of the themes and sub-themes are shown in Table 2. One of the included quotes makes reference to occupational health (OH) services. In the UK, there is only a legislative requirement to provide OH services amongst larger employers. Consequently, OH services paid for by the employer are provided to around 60% of workers. Services vary considerably but may involve telephone or face to face access to physicians, nurses or other allied health professionals who specialise in occupational medicine.

**Table 2:** Additional participant quotes to support the identified themes and sub-themes

Theme 1: CTR is not a ‘minor’ procedure		
*“I suppose actually just mentally preparing myself, because obviously, I’d never had any surgery done on a hand or a foot, or anything like that before. Obviously, you don’t realise beforehand how frustrating it’s going to be to not be able to use it, if that makes sense? I even struggled with going for a shower, trying to wash your hair and things like that. I had to get my partner, bless him, to wash my hair. It’s just mentally preparing yourself- To not be able to do as much as you would normally, but I suppose that’s the same for any surgery. I suppose I just didn’t prepare myself for what I could and couldn’t do.”* Fiona, animal technician (employed)		
*“A lot of people don’t realise, do they, how much is involved with carpal tunnel [surgery]. They think, “Oh, it’s just your hand. It’s just a minor op[eration].” But actually, it does affect you a lot in your working areas, wherever you are, whatever you do. They don’t realise how much it is going to affect the daily activities of living afterwards.”* Debbie, nurse (employed)		
Theme 2: Validation of the time taken off work		
i. Is the sick note a barrier for earlier return to work?	ii. It held more weight coming from the surgeon	iii. The patient role in the prescription of sick leave
*“Not really [I don’t recall any advice about returning to work]. Not that I remember. Only more about how much time to take off”* Amanda, administrator (employed)	*“At least I could give them fair warning, which was fine. And the fact that I had a doctor’s certificate. I had the surgery, and the surgeon said, “No, I will give a certificate straight off for 2 weeks anyway.” So I had warned the employers that 2 weeks [would be the] minimum.”* Alison, carer (zero hours contract)	*“Probably just be more steadfast in our own opinion because I felt, not intimidated, that’s the wrong word, I just thought, “Well, because they’re an expert, they know better than me.” I could see myself and I could feel myself that I wasn’t ready to use my hand and it didn’t feel as if I should have had the stitches out.”* Emma, optician (employed)
*“I just… Obviously took in my sick note that the hospital gave me and I just said I’ll keep in touch and see how we go.”* Peter, Mechanic (employed)		
Theme 3: Handling the return		
i. Making a graded return to work duties	ii. Travelling to work	
*“I was a bit anxious about coming back to work. I knew I still had trouble using my hand. I would have like a phased return to work. I don’t think they would have been supportive... I needed someone to sit down and say, ‘Look, [Emma] can’t come back full time. She needs to come in at 2:00 pm and go home at 4:00 pm’.” Whatever.* Emma, optician (employed)	*“I had it done over the weekend, and within a couple of days I was back driving, because I had absolutely nil pain from the wound. The pain that I used to get when driving was totally gone.”* Sarah, stable owner (self-employed)	
	*“Like, getting in the car, I didn’t drive for over 2 weeks... I didn’t feel happy to because my wrist felt, I don’t know, just not quite strong enough. I was worried. It’s alright if the roads aren’t busy and you could just go along, but if I had to react to something quickly, I didn’t feel comfortable with that. Yes. I was told, advised for 2 weeks not to [drive] and then to see how I felt after that.”* Amanda, administrator (employed)	
*“I was never forced into anything. It was always my decision as to whether I was happy or not. Certainly, my sergeants and inspectors have been very good and were just keen to do whatever is necessary to get me back out on the frontline again.”* Donna, police officer (employed)		

#### Theme 1: CTR is not a ‘minor’ procedure

It appeared that the level of functional disability experienced by the interviewees in the immediate post-operative period was unexpected, therefore the procedure was not as ‘minor’ as they had initially thought. All interviewees recalled experiencing difficulty with hand function and many reported that they had required assistance from their partners or children with activities of daily living. Showering and cooking were in particular described as problematic, as were dressing and tying shoelaces. None of the interviewees reported that they had made any prior plans for how to manage their daily activities after surgery. There seemed to be a pre-operative expectation that CTR would be a minor procedure, but this did not appear to fit with the participants’ experiences of their recovery. Despite this lack of preparation, many participants looked back on the immediate post-operative period with humour, recalling the unusual methods and strategies they had used to cope with having one hand out of action, as discussed by Debbie:

*“You don’t realise how much you depend on your hands until you can’t use them. I managed to adapt with having a shower and sticking my hand out behind the curtain (laughter). But washing hair and drying hair was an absolute nightmare. It didn’t happen properly. Cooking, yes, was a nightmare. I found I couldn’t lift a saucepan properly with my left hand. It wasn’t as strong as my dominant hand. And even cutting up your dinner, you really don’t realise. You do find ways to adapt in the end, but you just don’t realise how you rely on your dominant hand all the time. It was a good fortnight to be able to even grip a knife to cut anything properly. I just couldn’t grip it. It was too painful across the palm of the hand where the cut was, to grip the knife… But I think I would have prepared for it a bit more. Yes. Or even roped a friend in more to come and do things for me.”*

Debbie, nurse (employed).

In probing how the mismatch occurred between the anticipation that CTR would be a ‘minor’ procedure and the level of functional disability experienced, participants recalled receiving some information peri-operatively, but reported that this focused on wound management and avoidance of infection, rather than hand movement or function. Furthermore, the method of information delivery was often reported to be difficult to access, as suggested by Emma:

*“I was just told to keep it dry. No washing up. I was just told what I couldn’t do, rather than anything that might help me do day-to-day tasks… I think the exercises could have been given in a different way. I was just given a sheet of paper. It was in my pack, it wasn’t even pointed out to me. I found it in my pack. The trouble is in hospital, they give you lots of information, but it’s in a pack”.*

Emma, optician (employed).

Participants added that they felt that they were left to interpret general guidance for their own situation, and suggested that their clinicians could take a more proactive role in flagging up daily activities that might be difficult and suggesting ways to tackle this.

#### Theme 2: validation of the time taken off work

The second theme concerned the process of obtaining validation for taking time away from work. This related to official validation in the form of sickness certification, and also to an internal validation, as individuals experienced a need to justify to themselves that there was a valid reason to be away from work.

##### Is the sick note a barrier for earlier return to work?

Sickness certification was discussed by all employed participants. This was viewed as the formal process that allowed authorised work absence and was the method of communicating with employers about when the clinician had ‘permitted’ return to work. The language used by participants centred on the traditional *sick* note, which only recorded a prescribed duration of work absence, rather than its 2010 replacement, the *fit* note, which now includes sections for suggested activity modification to enable return to work [12]. It appeared that participants viewed the recommended time frame for work absence recorded on this document as a definite minimum period of absence. In effect, the fit/sick note could be perceived as a barrier, with participants only eligible to return to work after this prescribed time period. Importantly, this was the case for participants both with and without occupational sick pay. Interviewees appeared to trust their clinician that this was the correct thing to do in order to optimise their recovery. Having a certified period of work absence also appeared to validate their time off work, not just to the employer, but also to the individual themselves, as Fiona outlines:

*“He did sign me off for 3 weeks. He said, “Because of my work,” but obviously, after the 2 weeks, I went to see him and I had the stitches out… Yes. He said then, “You can return to work but on lighter duties.” He said he’s done the sick note for 3 weeks, so it was up to me really… I was thinking about going back to work after 2 weeks, but that’s just because you get a bit bored at home when you’ve got nothing to do. I’m glad I took the 3 weeks, because if I went back after 2 weeks, I would have done more than what I should have done… If your surgeon signs you off for a certain amount of time, I would take that, all of that time, to recover properly.”*

Fiona, animal technician (employed).

Unlike the employed participants, who returned to work after the timescale documented on the fit/sick note, those who were self-employed all reported returning to work earlier than had been verbally recommended. As might be expected, the reasons for this were primarily financial. All self-employed interviewees worked in roles with elements of heavy manual activity and reported return to work within 1–3 weeks of their surgery. None of these participants reported significant negative effects of earlier return to work, but on reflection, the majority felt that they had returned too soon. It is possible that the awareness that they had returned to work earlier than advised contributed to this reflection, as discussed by Alan below:

*“I went back to work as soon as I possibly could, you know, because no one’s going to pay me if I don’t earn money…. Probably a little bit too early, I did jump the gun a little bit, but now I’m okay, so it’s all good”.*

Alan, maintenance worker (self-employed).

##### ‘It held more weight coming from the surgeon’

The legitimisation of their period of work absence was discussed by several interviewees and formed an important part of their return to work experience. Participants appeared grateful when they received a fit/sick note from their surgical team. Some felt this ‘held more weight’ than a fit/sick note from their GP while others had been concerned that they would not be able to get an appointment with their GP to provide certification for their sick leave. It appeared that most employed interviewees felt that they needed strong justification for being off work and overwhelmingly, the surgeon was viewed as the optimal person to provide this justification, as illustrated by Emma below:

*“[The fit/sick note] was given to me straight away, so I didn’t have to ask for it. It did. That lasted the whole period. That was one of the most helpful things. Having the six-week note from the surgeon, rather than having to go to my GP. It held more weight, coming from the surgeon.”*

Emma, optician (employed).

After the initial post-operative period, a number of interviewees who worked in manual roles reported that they would have found a return to work interview or assessment beneficial at the end of their period of prescribed sick-leave. These individuals did not feel quite ready to return to work at this point, but seemed to feel unable to extend their period of sick leave. The key reason for this appeared to be that they felt a need for an external individual to guide and reassure them that additional work absence and/or job modifications were justified. Interestingly, in these interviews, they did not look to their surgeon to provide this information, rather someone from within their workplace, as described by Alison below. It appeared that these individuals were looking for someone with knowledge of their particular work role and pressures to be able to appropriately direct their return to work process and to endorse their belief that more time off was required.

*“I think, on reflection, had I had an interview before returning to work, or had I had some sort of occupational health check, I think that would have guided me. Had they said to me at that check then, “Well, [Alison], let’s give it another week,” I would have said, “Alright then.” Because I was being told officially, if you like. I’ve always been a little bit like that. I’m not the sort of person that will go off sick… You sort of get on with it, but then you realise that perhaps you should have given yourself another couple of weeks, I think. So that was it. There was no return to work interview or anything, which possibly in my previous employment I may well have had.”*

Alison, carer (zero hours contract).

##### The patient role in the prescription of sick leave

Recommended times to return to work or other functional activities, such as driving, were primarily viewed as prescribed time points, specified by the surgeon for the patient to follow. However, Vicky reported negotiating a shorter period of work absence when her fit note was being written. Interestingly, this was on the day of surgery, suggesting that Vicky may have decided when she would be able to return to work, in advance of any experience of her post-operative symptoms or functional ability.

*“I mean, to be fair, he tried to sign me off for 4 weeks, and I said, “How about one?” He said, “Well, let’s just say on light duties,” and gave me a sick note, do you see what I mean?... I said to him- because he laughed and he went, “Well, that’s what I would do,” So I said to him, “We’re both singing on the same hymn sheet, then.” I know some people would have been more than happy to go, “Yes, great, I have a month off.” But like he said to me, if you like your job, what’s the point? Do you see what I mean?”*

Vicky, secretary (employed).

While the large majority of participants highlighted the importance of following their clinician’s recommendations, one interviewee held an opposing view on the role of advice. For this individual, a 68-year-old self-employed gardener, advice from any ‘expert’ was not something to simply follow, but rather one consideration in a personal decision-making process.

*“Well, you’ve just got to play it by ear really. When it was all strapped up it was a bit awkward. I was advised not to work, but of course I did. I mean sod that. [I don’t care about that]. I mean it’s rather like accountants or anything else, you take the advice on board that they give you and adapt it to your own use.”*

George, gardener (self-employed).

#### Theme 3: handling the return

Two commonly reported sub-themes occurred as participants discussed handling the return to work process. The first was the need for a graded increase in hand function, while the second centred on travelling to work.

##### Making a graded return to work duties

After their initial period of sick leave, most participants described features of a graded return to work. The degree of modification varied: for some individuals, this meant taking longer to carry out their work activities, asking co-workers to assist with heavier tasks, or wearing a protective wrist splint. For other interviewees, there was a formalised structure involving the employer and/or occupational health clinicians. None of the participants saw return to work as part of their post-operative rehabilitation, rather the emphasis was on resuming work roles without causing pain or damage to the healing hand.

The outcome of a workplace risk assessment was discussed by one interviewee. Fiona was very happy with her return to work and the processes in place to guide this, however it was interesting that she described the situation in terms of a prescribed return to work programme, rather than an active dialogue between herself and her employer.

“*I had to do a risk assessment with the health and safety officer, my supervisor there. I wasn’t allowed to do any cleaning for at least 3 to 4 weeks, I think it was… Yes, because of the heaviness, because of the actual manual work involved, they didn’t want me to go back to that. Even when I did the feeding, they said, “Oh, you can try feeding,” because sometimes, you’re still lifting heavy things. I wasn’t allowed to lift panels. I could only lift feed buckets that I felt I was comfortable to*”.

Fiona, animal technical (employed).

The return to work experience described by other interviewees was more informal and self-directed, but still had elements of gradually resuming normal activities, as Peter, who worked as a mechanic, discusses below:

“*I was planning just to go back and go on the computer and do the invoices and things, but that didn’t work out, so I just did light stuff that I could do with my right hand. If there was anything that I needed to move or lift then someone else moved or lifted it for me*.”

Peter, mechanic (employed).

Some interviewees reported hand/wrist pain associated with return to work. They linked the pain to specific work tasks, such as using hand-held machinery, opening jars and pushing down on a stapler. For many this served as a reminder to use the hand less forcefully or to modify the activity. Other interviewees reported that a lack of dexterity and/or strength were their main problems on return to work. This meant that there were certain activities they felt physically unable to do when they first returned. Interviewees described breaking down their work role into activities that they could and couldn’t do, as Debbie recalled:

*“I mean, I was there in body and useful to do some things, but I couldn’t do the full job for a start. I couldn’t grip, to be quite honest. I couldn’t do the things like taking out stitches and things like that at work. I couldn’t grip the scissors properly.”*

Debbie, nurse (employed).

Regardless of the job role or duration of work absence, all participants recognised a need to modify their work activities to some extent when they first returned. Interestingly only one participant reported receiving advice about how to return to work from their surgical team. Charlotte reported being advised to “go back on light duties; answering the phone and typing” (Charlotte, postal worker). In contrast, the majority of interviewees were surprised by an absence of tailored return to work advice from their surgeon. Interviewees recalled that the principal advice from their surgeons was how long to take off work. For some, this led them to feel that they had returned to work too soon. This was particularly the case for interviewees with manual roles (i.e those who potentially required greater work modifications) and those without a formal work-based return to work process (i.e those without other ‘official’ sources of targeted return to work advice). This experience is illustrated by Emma’s quote below, which revealed that she had to initiate the discussion about work and found the guidance unclear.

*"I didn’t have any advice on what to do when I went back…. None of them talked about work, unless I asked. I said to my surgeon, “What should I be doing?” He said, “Just treat it as normal now. Just use your hand as normal, but take it slowly.” I thought, “What does that mean?”*

Emma, optician (employed).

##### Travelling to work

The need to drive was a limiting factor for return to work for a large group of interviewees.

Many reported that a lack of public transport made it extremely difficult to get to work if they were not able to drive; however, Charlotte, who was reliant on public transport to travel to work, also found this difficult in the early post-operative stages.

*“[It was] a little bit of a struggle when I get- I use the public transport, because- I mean you’ve got only one hand and then when you want to get on and get off, it’s quite difficult.”* Charlotte, postal worker (employed).

Two recommendations were commonly recalled relating to return to driving: first, that they had been advised to return after removal of sutures, and second, to return from 2 weeks after surgery. In practice, these are similar time points, as sutures are usually removed at 10–14 days. As with the return to work time points discussed above, most interviewees reporting strictly adhering to the advice they were given: *“Obviously I couldn’t drive until I had the stitches out”* (Vicky, secretary). In comparison, several interviewees reported driving at earlier time points, as illustrated by Alan:

*“I mean, he said to me, a week before I drive, and I drove home. But I had an automatic at the time, so it didn’t bother me”.*

Alan, maintenance worker (self-employed).

A number of other interviewees also raised the different requirements for driving depending on the side of surgery or the type of car. One interviewee, who had CTR to both hands within the REACTS study period, highlighted the main perceived differences:

*“But the left was worse than the right, because of course I’ve got a manual car, so the gear change was particularly- I wouldn’t say difficult. I wouldn’t say I would have gone on a long journey, I wouldn’t like to have done an emergency stop. Yes, so gear changing was probably challenging, I think the word is (laughter). I had to use two hands to pull the handbrake on when I had the left done, but the right, because - you don’t use your right, you only use it on the steering wheel.”*

Vicky, secretary (employed).

## Discussion

Through the use of qualitative interviews, we have gained an understanding of patients’ experiences of returning to work after CTR and provided insight into the factors shaping decision-making for return to work. Three key themes were identified: the perception that CTR is *not a ‘minor’ procedure*; the desire for both internal and external *validation of the time off work*; and reflections on *handling the return* to work. These findings highlight important topics for clinicians to discuss with their CTR patients and provide context for the development of specific return to work guidance. We did not identify any explicit barriers or facilitators for return to work; instead the picture appeared more complex. Interviewees appeared to be seeking specific information from the surgeon regarding when they should return to work and how to make a graded return (potential facilitators for return to work), yet when a period of work absence was documented on a fit note, no interviewees returned to work before that time point (a barrier to earlier or self-directed return). A similar scenario was observed for return to driving. These findings suggest that there is a need to be mindful of both the potential positives and negatives of any change in return to work strategy after CTR, and that there is an expectation for surgeons to be able to understand the work demands of many very different work roles. The majority of interviewees appeared to be looking for specific authorisation regarding timings and strategies for returning to their work duties with an expectation that their surgeon could provide definitive information. Wound healing after CTR has been described in studies comparing different suture materials. Macfarlane et al. found that 2 weeks after CTR, 66% of participants had achieved wound healing with mild bruising (Southampton grade 1) and by 6 weeks 98% had achieved complete healing (Southampton grade 0) [13]. However wound healing in the intervening period was not captured and therefore these findings do not easily translate to appropriate timescales for resuming different functional activities after CTR. Our previous survey of UK hand surgeons and hand therapists identified that these clinicians recommended a wide range of return to work times for the same occupational duties and reported divergent views on whether it was safe for patients to return to work before suture removal [14].

We found that interviewees had largely underestimated the immediate functional impacts of CTR, but the reason for this mismatch between expectation and experience was not clear. Some participants reported that they felt that they had been given insufficient information about this aspect of their recovery, while others suggested that there was a general perception of CTR as a ‘minor’ procedure, which may have shaped expectations at a more sub-conscious level. In healthcare nomenclature, CTR often is termed a ‘minor procedure’ as demonstrated by the National Institute for Health and Clinical Excellence surgery grades [15]. This terminology is misleading because ‘minor’ can be interpreted in a range of ways and may be understood differently by patients and clinicians. The reported reduced ability to grip and/or weight-bear through the hand after CTR, coupled with the described recommendations to keep the wound dry until removal of sutures had an important impact for the majority of interviewees. The level of complexity of the procedure did not seem to equate to the level of impact felt by the patients and this contributed to a reported lack of preparedness in the immediate post-operative period. Previous qualitative interviews with CTS patients found that their CTS symptoms detrimentally affected their quality of life and their hope was for CTR surgery to resolve this [16]. If high expectations for the benefit of surgery are common among CTS patients, unexpected post-operative problems with function may be particularly distressing. This could potentially be improved by clinicians communicating the likely post-operative impacts of the surgery and by suggesting strategies to help manage ADLs in the immediate post-operative period. Access to relevant information, including suggestions for how to manage at home post-operatively, has been identified as essential for positive patient experiences following other elective surgeries [17].

The concept of information provision was also raised in the third theme (handling the return) with many interviewees highlighting that they needed more advice about how to return to their work, including information that they could share with their employers. It seemed that employed interviewees saw themselves as information conduits to deliver medical recommendations between their surgeon/GP and employer, a finding that has also been reported in a qualitative study of employees returning after workplace injury [18]. It is not clear whether this is the optimal model of care for CTR patients (or indeed any patients), and future work might explore whether using the additional comments section of the fit note could be a way of providing the targeted information that the interviewees appeared to be seeking [12].

The interviewees placed a strong emphasis on the need for formally authorised work absence *(validation of time off work)*. This might be expected given that formal authorisation is often required by employers and for statutory sick pay, but for many interviewees this validation was also to justify to themselves that there was a real need to take time off work. It was interesting that the current ‘fit note’ system for authorising work absence was never referred to as such, rather as its previous incarnation, the ‘sick note’, or as simply a ‘certificate’ or ‘being signed off’. Furthermore, the fit note appeared to have been used in the manner of a sick note to indicate a period of ‘prescribed’ work absence, rather than to indicate work adjustments under which the patient may be fit for return. A similar finding was reported in a systematic review of fit note use in the UK, which found that only a small minority of patients treated in primary care received the recommendation that they ‘may be fit’ for work with structured advice and/or comments on the functional effects of their condition [19]. Our recent survey of hand surgeons and hand therapists found that those who treated approximately 1–2 CTR patients each week recommended earlier return to work times than those who saw this patient group less frequently [14]. This perhaps suggests that those with more experience in treating CTR patients believe that earlier return to work (i.e. before the patient is 100% fit) is not detrimental to recovery. In the current study, interviewees appeared deterred from returning to work before the period of time documented on their fit/sick note due to the assumption that this would be going against clinical advice. This suggests the potential power that appropriate evidence-based return to work advice could have if delivered and documented by the surgeon. Receiving authorised time off work appeared to be beneficial to the interviewees’ experience, but for some adherence to this prescribed time period became a barrier to earlier return to work. The current NHS guidance for patients, available on the ‘common health questions’ NHS website is potentially ambiguous [20]. It states that “You should go back to work as soon as you feel able to and, with your employer’s agreement. This may be before your fit note runs out.” However, two paragraphs later it states “You should not go back to work before the end date on your fit note if your doctor has advised that you should stay off work for the full period covered by the fit note”. In practice, it may not be clear to the patient which of these statements best applies to them. It is conceivable that the fit note could be better used to provide clarity for patients after CTR and that clinicians could better empower their patients to manage their post-operative rehabilitation and return to work without a focus on rigid timelines.

All interviewees discussed a need to modify their work activities on initial return to work. This ranged from a formalised graded return programme to more informal situations, such as asking co-workers for help with certain tasks. Only one interviewee recalled receiving information from their surgeon with suggestions for how to modify their work. In addition to the recommended timescale for returning to work, interviewees reflected that practical suggestions for how to build up activities would have been helpful and this may have reduced uncertainty regarding resuming work activities. This raises the question of whether surgeons can really be expected to understand the intimacies of the multitude of job roles. We propose that a coordinated approach is required, with the surgeon focusing on the clinical recovery from the surgery, aligned with the patient or employer’s understanding of their work duties and available modifications. The current study found that patients appeared to value most highly the recommendations that they received from their surgeon. Therefore, in the absence of explicit evidence regarding when it is safe to return to different functional activities after CTR, it would be useful to explore a consensus among hand surgeons. This would enable the provision of consistent and appropriate advice for CTR patients regarding return to general work-related and functional activities.

### Limitations

This interview study was designed, conducted and reported in accordance with the COREQ checklist [21]. However there are still a number of limitations. Firstly, individuals were only invited to participate in an interview after completing the associated REACTS cohort study and may therefore have differed from those who declined to participate or were lost to follow-up. Furthermore, the surgeons involved in the REACTS study may have given greater consideration to providing work-related advice than the wider hand surgery population, given the nature of the research. However, this recruitment approach was necessary to allow for a purposive sampling strategy.

The second limitation is that the proportion of male interviewees was lower than the proportion invited, and lower than across the cohort as a whole. This had the potential to over-represent the experiences of women. To address this, any marked differences in gender were explored in the early stages of the analyses and sex-effects were not apparent. Interviewees had been treated in a range of different healthcare settings (primary care, secondary care and private healthcare) and had encountered different CTR patient pathways; the research team are confident that the interviewees illustrated a broad range of experiences and that interviewing was continued until data saturation was met.

Thirdly, the interviewer was a practising physiotherapist, specialised in hand therapy. While none of the participants were known to the interviewer in a clinical capacity, it is possible that knowledge of the interviewer’s background may have influenced the interviewees’ responses. The potential impact of this was discussed during data analysis. In addition, steps were taken to ensure that the analysis was not conducted solely by clinicians working with CTR patients. This included the involvement of patient advisors and an experienced qualitative researcher without clinical or academic experience of CTS, CTR, hand therapy or hand surgery (CB).

## Conclusions

The key themes identified from this interview study suggest that there is a desire for more information explaining *how* to return to work and function after CTR. Patient experiences may be improved by clinicians (most notably the surgeon) communicating the likely short-term functional impacts of the CTR procedure and strategies to assist with this; initiating a dialogue with patients to discuss their work, with examples of how the individual might grade their return (this may be documented on a fit note); and by providing sufficient information to empower patients to be confident in their own decision-making regarding return to work and function. Future research should focus on establishing evidence-based guidance to inform return to different types of work after CTR and on understanding how best to engage clinicians, patients and employers with this guidance. The impact of any guidance on patient experience and return to work outcomes should also be evaluated.

## Additional file

Additional file 1: Qualitative Interview Topic Guide. (PDF 243 kb)

## Abbreviations

CTR: Carpal tunnel release
CTS: Carpal tunnel syndrome
NHS: National Health Service
NIHR: National Institute for Healthcare Research
REACTS: Return to employment after carpal tunnel release surgery
