# Supplementary material for: Return to work after carpal tunnel release surgery: a qualitative interview study
Source: BMC Musculoskelet Disord. 2019 May 22;20:242. doi: 10.1186/s12891-019-2638-5 (PMC6530142; doi:10.1186/s12891-019-2638-5)
Supplement: Supplementary file 1 — Qualitative Interview Topic Guide. (PDF 243 kb) [file 12891_2019_2638_MOESM1_ESM.pdf]

Qualitative Interview Topic Guide

Check agree to audio recording
Assure confidentiality

Introduction
The aim of this stage of the REACTS study is for me to talk to individuals who had carpal tunnel release surgery. I want to talk to you about your experience of the surgery and of returning to work afterwards. There are no right or wrong views. I am interested in hearing about your experience.

1. Can I start by asking: how does your work involve you using your arms and hands day-to-day?
   - *Prompt time schedule and breaks*
   - *Prompt repetitive activities – duration*
   - *Prompt heavy loads – frequency and duration*
   - *Prompt computer use - duration*
   - *Prompt any change in role as result of CTS/CTR*

2. Please tell me a bit about your hands and wrists felt before you had surgery?
   - *Prompt impact on work*
   - *Prompt impact on function and social activities*
   - *Prompt bilateral/unilateral*

3. How do your hands feel now?

4. How did you decide to have the carpal tunnel release surgery?
   - *Prompt clinic experience*
   - *Prompt referral process*
   - *Prompt pre-operative information about expected outcomes*
   - *Prompt any discussion about work*
   - *Prompt trial of splints/injections – how did they work for you?*

5. What sort of information were you given about your surgery and going back to work afterwards?
   - *Prompt pre-operative information and from whom*
   - *Prompt sick note/fit note – from whom, and any extension to the initial note*
   - *Prompt other sources of information (internet, friends and family)*
   - *Prompt satisfaction with information/advice provided*
   - *Prompt awareness of potential for complications eg infection, prolonged scar pain, weakness that could affect return to work*
   - *Prompt any other information that would have been useful*
   - *Prompt comments on the format of the information*

6. What medical treatment did you receive after your surgery?
   - *Prompt removal of sutures*
   - *Prompt physio/OT/splint*
   - *Prompt any contact with GP or occupational health*

- *Prompt treatment for complications*

7. How did you decide when to return to work?
   - *Prompt clinical signs/advice given*
   - *Prompt – how confident in using hand*
   - *Prompt amended duties*
   - *Prompt altered hours*
   - *Prompt occupational health*
   - *Prompt RTW criteria specified by employer*
   - *Prompt any contact with workplace whilst off work*
   - *Prompt driving/transport*
   - *Prompt financial*
   - *Prompt co-workers*

8. Tell me about your return to work – and how it went?
   - *Prompt anything that was particularly helpful for you when returning to work?*
   - *Prompt anything about your job or workplace that made it harder for you to return to work?*
   - *Prompt hand dominance*
   - *Prompt boss/co-workers*
   - *Prompt job satisfaction*

9. Do you think you were 100% fit to do your job when you returned to work?
   - *Prompt what percentage 'fit' do you think you were?*
   - *Prompt components of your job could you do despite not being 100% fit?*
   - *Prompt anything you couldn't do?*
   - *Prompt thoughts about return for different jobs*
   - *Prompt what where your employer's views – amended duties*

10. While off work how did you manage with your home or family commitments?
    - *Prompt washing, showering, personal care*
    - *Prompt cooking, shopping*
    - *Prompt assistance from others*
    - *Prompt not doing all usual activities*

11. Now, with the benefit of your experience, what advice would you give about returning to work to a friend who needed a carpal tunnel release?

12. Is there that you would like to add about returning to work after your surgery that we haven't discussed?

Thank you
Assure confidentiality
